# Short neuropeptide F signaling regulates functioning of male reproductive system in *Tenebrio molitor* beetle

**DOI:** 10.1007/s00360-020-01296-z

**Published:** 2020-08-04

**Authors:** Paweł Marciniak, Arkadiusz Urbański, Jan Lubawy, Monika Szymczak, Joanna Pacholska-Bogalska, Szymon Chowański, Mariola Kuczer, Grzegorz Rosiński

**Affiliations:** 1 Department of Animal Physiology and Development, Adam Mickiewicz University, Poznań, Uniwersytetu Poznańskiego 6 Street, 61-614 Poznan, Poland; 2 HiProMine S.A, Poznańska 8 Street, 62-023 Robakowo, Poland; 3 Faculty of Chemistry, University of Wrocław, F. Foliot-Curie 14 Street, 50-383 Wroclaw, Poland

**Keywords:** Short neuropeptide F, Reproduction, Testes, GPCR, Beetle, *Tenebrio molitor*

## Abstract

Neuropeptides of short neuropeptides F family (sNPF) have been identified in various arthropods. They are pleiotropic neuromolecules which so far have been mainly associated with regulation of feeding and metabolism, as well as growth and development, locomotion, circadian rhythm or learning and memory. Here, we describe the effects of Tenebrionid sNPF peptide (SGRSPSLRLRFa) on various aspects of the male reproductive physiology in the *Tenebrio molitor* beetle. We identified in silico the putative sNPF receptor Tenmo-sNPFR. Based on RT-PCR technique, it was shown that the receptor might be present in the male reproductive tissues of this beetle. The analysis of receptor amino acid sequence showed that it is similar to other beetle sNPFRs, as well as other insect species, and belongs rhodopsin-like G-protein-coupled receptors (GPCRs). Injections of Trica-sNPF and its shorter form Trica-sNPF_(4–11)_ caused differentiated effects in *T. molitor* male reproductive tissues. After 24 h post injections, the peptides decreased the concentration of the soluble protein fraction in testes of 4- and 8-day-old beetles as well as the dry mass of these organs but only in 8-day-old individuals. The same effects were shown with regard to accessory glands. Both peptides decrease the concentration of the soluble protein fraction but do not affect the dry mass of this organ. Furthermore, injections of Trica-sNPF at the 10^–7^ M concentration decrease the total sperm number in the reproductive system. Surprisingly, the same concentration of the shorter form, Trica-sNPF_(4–11)_ increased the sperm number. It was also shown that both peptides in different manner influence contractions of ejaculatory duct. The data presented in this article give new evidence that sNPFs are involved in the regulation of reproductive events in beetles, which might be the part of a larger neuropeptide network combining feeding, growth and development with the physiology of reproduction.

**Electronic supplementary material:**

The online version of this article (10.1007/s00360-020-01296-z) contains supplementary material, which is available to authorized users.

## Introduction

Neuropeptides are diverse neuron-derived peptides with neuromodulatory, neurotransmitter or hormonal functions. Most neuropeptides act via G protein-coupled receptors (GPCRs) (Hauser et al. 2006). These neuromolecules regulate various aspects of physiology, including growth, metabolism, and reproduction. Apart from juvenile hormone (Wijesekera et al. 2016; Wilson et al. 2003) little is known about the hormonal and neurohormonal regulation of the functioning of the male reproductive system in insects (Klowden 2008). Thus far, only few studies have been performed on insects (Marciniak et al. 2017; Rankin et al. 2009; Van Wielendaele et al. 2013).

One of the peptide group which might be involved in the regulation of reproduction in insects is short neuropeptides F family (sNPF). sNPFs are short neuropeptides with a chain length of 8–12 amino acids that have a typical *C*-terminal consensus sequence M/T/L/FRFa (Fadda et al. 2019). Due to the issues with nomenclature during their discovery they were included into neuropeptides F family, but they are evolutionary distant from true/long neuropeptides F (Nӓssel and Wegener 2011). However, it is now clear that both groups originated from the common ancestor of protostomes and deuterostomes (Fadda et al. 2019).

First sNPF (at the beginning designated as head peptide), which is not a member of neuropeptide F family, was identified in the midgut of *Periplaneta americana* (Veenstra and Lambrou 1995) and later in the brain of the beetle *Leptinotarsa decemlineata* (Spittaels et al. 1996). Since that time, a huge number of sNPFs sequences have been identified in various insects, i.e., fly *Drosophila melanogaster*, mosquito *Aedes aegypti* and moth *Bombyx mori* (Nӓssel and Wegener 2011). In beetles, they were identified in few other species including *Tribolium castaneum*, *Nicrophorus vespilloides*, *Dendroctonus ponderosae* and *Carabus *sp. (Pandit et al. 2018; Ragionieri and Predel 2019) and also predicted in other beetles (Pandit et al. 2019; Veenstra 2019). Analysis of precursor structure showed that in most beetle species it contains only one mature peptide. For example, in *T. castaneum* one peptide Trica-sNPF (SGRSPSLRLRFa) together with its truncated form Trica-sNPF_(4–11)_ (SPSLRLRFa) were identified (Li et al. 2008).

In insects, it has been shown that sNPFs are mainly involved in regulation of feeding (Fadda et al. 2019), as well as in other physiological processes, including reproduction and development (Nӓssel and Wegener 2011). Gonadotropic properties of sNPF were shown only in locusts. Single injection of sNPF leads to accelerated egg development, whereas prolonged injections stimulate ovarian development and increase the level of circulating vitellogenin in the haemolymph (Cerstiaens et al. 1999). In beetles, physiological properties of sNPFs have been demonstrated at first in *L. decemlineata*, where sNPFs level drop down in diapausing individuals suggesting their role in development (Huybrechts et al. 2004). In *Tenebrio molitor*, these peptides also delay the moulting process of larvae, accelerate pupal moulting and increase growth in pre-starved and non-starved larvae, which again shows that they regulate growth and development in beetles (Marciniak et al. 2013). Further studies with *T. molitor* have shown that sNPFs inhibit the oviduct muscle contractility (Marciniak et al. 2013) and stimulate the contractions of the ejaculatory duct in this species (Marciniak et al. 2017).

As most of the neuropeptides, sNPFs also act via G-protein-coupled receptors (Audsley and Down 2015). GPCRs have seven α-helical transmembrane domains and are, therefore, also called seven transmembrane (7TM) receptors and are one of the most common and important molecules in living organisms (Hauser 2008). The complete set of GPCRs for neuropeptides were first described in *T. castaneum* (Hauser 2008).

In the present study, the effects of Trica-sNPF and its truncated form on various aspects of male reproductive physiology in the *T. molitor* are analyzed. We here focused on effects of these treatments on the dry mass of testes and the accessory glands together with their soluble protein concentrations. Furthermore, we predicted the sNPF receptor (sNPFR) sequence in *T. molitor* and its transcript distribution to evaluate whether the observed effects of peptide injections are direct, due to ligand receptor interactions or indirect via other hormones.

## Materials and methods

### Insects

*T. molitor* adult males were reared according to a previously described procedure (Rosinski et al. 1978). For all injections males in two time points (4- and 8-day-old) were collected. Size of all individuals (length of elytra) was measured to exclude correlation of this parameter with observed changes in tested parameters.

### Peptides

Peptide Trica-sNPF (SGRSPSLRLRFa) and its truncated form Trica-sNPF_(4–11)_ (SPSLRLRFa) were synthesized according to the Fmoc procedure, as described previously (Lubawy et al. 2018; Marciniak et al. 2008). The purity of peptides was checked by HPLC and found to exceed 98% in each case. The peptides were dissolved in physiological saline appropriate for beetles (274 mM NaCl, 19 mM KCl, 9 mM CaCl_2_) to yield a stock solution of 10^−3^ M, and stored at − 20 °C for further use. Working dilutions were prepared from a stock solution in a physiological saline. In all in vivo bioassays, insects were injected (2 µL) with two concentrations of each peptide, 10^−7^ M or 10^−5^ M to reflect physiological (10^−8^ M) and pharmacological (10^−6^ M) concentrations of peptide in the beetle. The total haemolymph volume of *T. molitor* is about 20 µL (Marciniak et al. 2017).

### Transcriptome sequencing, database search and sequence comparison

Transcriptomic data from *T. molitor* [BioProject: PRJNA608239; Sequence Read Archives (SRA): SRR11184806] were obtained after Illumina Hiseq sequencing of total RNA extracted from the brain and retrocerebral complex of adult beetles performed at Beijing Genomics Institute (Shenzhen, China). After initial filtering of low quality reads and adaptor removal, clean reads were de novo assembled using Trinity and used for local tblastn with receptor sequence from *T. castaneum* (Hauser 2008) to find *T. molitor* short neuropeptide F receptor sequence.

The predicted protein sequence of the Tenmo-sNPFR ORF (open reading frame) was analyzed for the presence of putative transmembrane regions with the software programs PSIPRED—MEMSAT (https://bioinf.cs.ucl.ac.uk/psipred/) (Nugent and Jones 2009). Phosphorylation, glycosylation and palmytoylation sites were predicted, respectively, with NetPhos 3.1, NetNGlyc 1.0 and CSS-PALM 4.0 (https://www.cbs.dtu.dk/services/NetPhos/, https://www.cbs.dtu.dk/services/NetNGlyc/ and https://csspalm.biocuckoo.org/online.php) (Blom et al. 2004; Ren et al. 2008). In addition, two-dimensional representation of the receptor was created in TOPO2 (Johns S.J., TOPO2, transmembrane protein display software, https://www.sacs.ucsf.edu/TOPO2/).

Protein sequence alignment of Tenmo-sNPFR with other Coleopteran sNPFRs was performed with Clustal W (https://embnet.vital-it.ch/software/ClustalW.html) and includes *T. castaneum* (XM_015977710.1), *Asbolus verrucosus* (A0A482WBQ0), *D. ponderosae* (XM_019906309.1), *Hylobius abietis* (Pandit et al. 2018), *L. decemlineata* (XM_023163588.1), *Diabrotica virgifera virgifera* (XM_028295643.1), *Agrilus plannipenis* (XM_018479113.1), *Aethina tumida* (XM_020011983.1), *Ontophagus taurus* (XM_023053562.1), *Anoplophora glabripennis* (XM_018716007.2) and *N. vespilloides* (XM_017924316.1). To compare Tenmo-sNPFR sequence with other insect species and human prolactin-releasing peptide receptor (PrRPR) the following sequences were used: NP_001262086.1 for *D. melanogaster*; XP_021705044.1 for *A. aegypti*, ALP48446.1 for *B. mori* and NP_004239.2 for human PrRPR. All alignments and similarity analysis were visualized with usage of Jalview and Ugene softwares.

### Receptor transcript distribution

Transcript profiles of Tenmo-sNPFR were determined by reverse transcriptase PCR (RT-PCR) in various tissues of 4-day-old males. RT-PCR was performed according to a modification of the method described by Marone et al. (2001). Suitable tissues (brain, testes, ejaculatory ducts and accessory glands) after dissection were transferred to 150 µL of RNA lysis buffer (Zymo Research, USA) and homogenized for 3 min using a pellet homogenizer. The homogenized tissues were immediately frozen in liquid nitrogen and then stored at −80 °C. A Quick-RNA^®^ Mini Prep kit (Zymo Research, USA) was used for RNA isolation. Quantification and verification of sample quality was done by the spectrophotometer (Synergy H1 Hybrid Multi-mode Microplate Reader, BioTek, USA). Reverse transcription of the same amount of isolated RNA (200 ng) to cDNA was accomplished using the RevertAid™ Reverse Transcriptase kit (Thermo-Fisher Scientific, USA) according to the manufacturer’s protocol. PCR analyses were conducted using a T100™ Thermal Cycler (Bio-Rad, USA). The primers were designed based on sequences of Tenmo-sNPFR using Primer3 software (Untergasser et al. 2012). The primer pair was created to amplify fragments of 143 bp with the following sequences Fw 5′-ACTTCTACCACCAGATCA-3′ and Rev 5′-CACCTGTTTGAACTCCTT-3′. The primers were synthetized by the Institute of Biochemistry and Biophysics of the Polish Academy of Sciences (Warsaw, Poland). PCR was performed in a 10 µL reaction volume containing 3.95 µL of DNase⁄RNase-free water, 1 µL of DreamTaq™ Green Buffer (Thermo-Fisher Scientific, USA), 1 µL of 2 mM dNTP, 1 µL of 10 µM forward primers, 1 µL of 10 µM reverse primers, 0.05 µL of DreamTaq™ DNA polymerase (Thermo-Fisher Scientific, USA) and 2 µL of cDNA. The obtained products were analyzed by electrophoresis using a 2% TAE agarose gel stained with ethidium bromide. The Quick-Load Purple 100 bp DNA Ladder (New England BioLabs, USA) was run on each gel. Photos of the agarose gels were taken using ChemiDoc™ Touch (Bio-Rad, USA). Minimum of five biological and three technical repeats were made. To confirm our results, the bands were sequenced with BigDye Terminator v3.1 on an ABI Prism 3130XL Analyzer (Applied Biosystems, Foster City, CA, USA) according to manufacturer's protocols by the Molecular Biology Techniques Laboratory (Faculty of Biology, Adam Mickiewicz University in Poznań) and compared with transcriptomic data. “No template control’’ and “no RT control’’ reactions were included in the analysis to ensure that there was no foreign DNA or genomic DNA contamination.

### Determination of soluble protein concentration in the accessory glands and testes

The bioassay was performed according to the previously described procedure (Marciniak et al. 2017). The accessory glands and testes were collected from 4- and 8-day-old beetles (referred as younger and older, respectively) injected with a physiological saline (control) or peptide. Each injection was performed 24 h prior to tissues collection. During that time all males were kept separately from females. For each group of tissue samples, whole bean-shaped accessory gland and testes (1 testis/insect; second was used to determine the dry mass described below) were collected and immediately transferred into 50 µL ice-cold physiological saline. Five tissues collected from five individuals we regarded as one replication. Next, tissues were homogenized on ice for 5 min using a cordless Pellet pestle motor (Kimble Chase, USA). The homogenate was centrifuged at 10,000×*g* for 5 min and the supernatant was transferred into a new tube. The protein concentration in the supernatant was determined using infrared spectrometer Direct Detect (Merck, Germany).

### Evaluation of the dry mass of the accessory glands and the testes

The bioassay was performed according to the previously described procedure (Marciniak et al. 2017). Testes and accessory glands were collected separately from 4- and 8-day-old beetles (referred as younger and older, respectively) injected with physiological saline or peptide. Each injection was performed 24 h prior to the collection of the tissues. During that time all males were kept separately from females but due to olfactory stimulation of maturation of males reproductive system, females were kept in the same breeding chamber. For each group of tissue samples, whole accessory gland or testes from five individuals (1 testis/insect; second was used to evaluate soluble protein concentration described previously) were collected. After isolation each organ was carefully washed in demineralized water. Organs collected from five individuals were regarded as one repetition. Each sample was weighed and then dried using Christ RVC 2–18 rotational vacuum concentrator system for 45 min at 60 °C. After drying, the samples were weighed and their dry mass was determined.

### Total sperm cells number bioassay

Total sperm cells number was assessed in the testes and *vas deferens* including seminal vesicles of the males based on the method described earlier (Marciniak et al. 2017). In brief, after dissections testes were transferred to 250 µL of physiological saline. Using microsurgical tweezers, testes were opened and spermatocytes were released. Subsequently, a suspension of spermatocytes in physiological saline was vortexed and transferred (15 µL of the suspension) to a glass slide. To better estimate total sperm cell counts, the procedure was performed in duplicate in all cases. The sperm cell suspension was dried in air for 30 min and fixed in a solution of 4% paraformaldehyde in physiological saline for 15 min. Fixed spermatocytes were stained for 10 min using DAPI (4,6-diamidino-2-phenylindole), washed and mounted with mounting medium (90% glycerol, 2.5% DABCO, PBS). Samples were examined with a Nikon Eclipse TE 2000-U fluorescence microscope equipped with a Nikon DS-1QM camera. For each specimen, photos of seven random regions were taken. For the determination of the total sperm cells number an algorithm based on the mean number of spermatozoids in the pictures, the specimen’s surface area and the magnification of the microscope [the sum of spermatocytes nuclei visible on 7 photos × (total suspension volume/the volume of suspension transferred to a glass slide) × (surface area of specimen/surface of photos area)] was used. The sperm cells presented on photos were counted with the computer programme ImageJ (ver. 2).

### Ejaculatory duct contraction bioassay

The ejaculatory duct bioassay was conducted as described previously using a video microscopy technique (Marciniak et al. 2011; Marciniak and Rosinski 2010). In brief, isolated ejaculatory duct on Sylgard filled chamber were placed under the Olympus SZX12 stereomicroscope equipped with a SD30 camera. An open perfusion system was used, with an injection port (for peptides) 70 mm above the superfusion chamber. The organ was subjected to a constant perfusion with fresh saline at the rate of about 140 mL/min. All tested samples were applied at the injection port with a Hamilton syringe. Many pulse applications of samples could be sequentially assayed in a single preparation. After the initial 15 min stabilization, the activity of the isolated organ was recorded for 2 min. Next, the peptide was applied and the ejaculatory duct activity was recorded for a further 2 min. The video recordings of superfused organs were analyzed with the edge tracking software (AnTracker) to create a trace of the movement of the side edge of the organ.

### Analysis of males fertility

The evaluation of male fertility after neuropeptides injection was based on determination of eggs hatchability. In this experiment, 4- and 8-day-old males and 4-day-old females were used. Females age selection was related to the fact that duration of ovary maturation in females of *T. molitor* last four days. Tested males were injected with tested peptides, in two concentration (10^–7^ and 10^–5^ M) or physiological saline (control), 24 h before experiment. In each biological repetitions, five males and five females were placed in small, transparent plastic box (size: 9 cm × 7 cm × 5 cm). In each box, the same amount of flour and carrot was provided. Then, the boxes with males and females were left in breading chamber for 24 h. After that, males were removed and females were kept in the boxes for the next 72 h. After this time, number of eggs laying at the bottom of box and in the flour was counted. To provide stable temperature and air humidity, boxes with eggs were kept in plastic container with a saturated sodium chloride solution. The plastic containers were placed in breading chamber. Number of hatched larvae were checked each day for a period of 14 days or until all larvae hatched. To avoid cannibalism, young larvae were removed from boxes. Based on this observation, the percentage of hatched egg was estimated. For each of research variants, no < 5 biological repetition were performed.

### Statistics

All statistical comparisons (nonparametric *t* tests) were performed with usage of Graph Pad Prism 6 software (AMU licence). Results were considered statistically significant with *p* < 0.05. Prior to the analysis Shapiro–Wilk normality test was done for all of the groups.

## Results

### Analysis of *T. molitor* sNPFR sequence

**Fig. 1:**
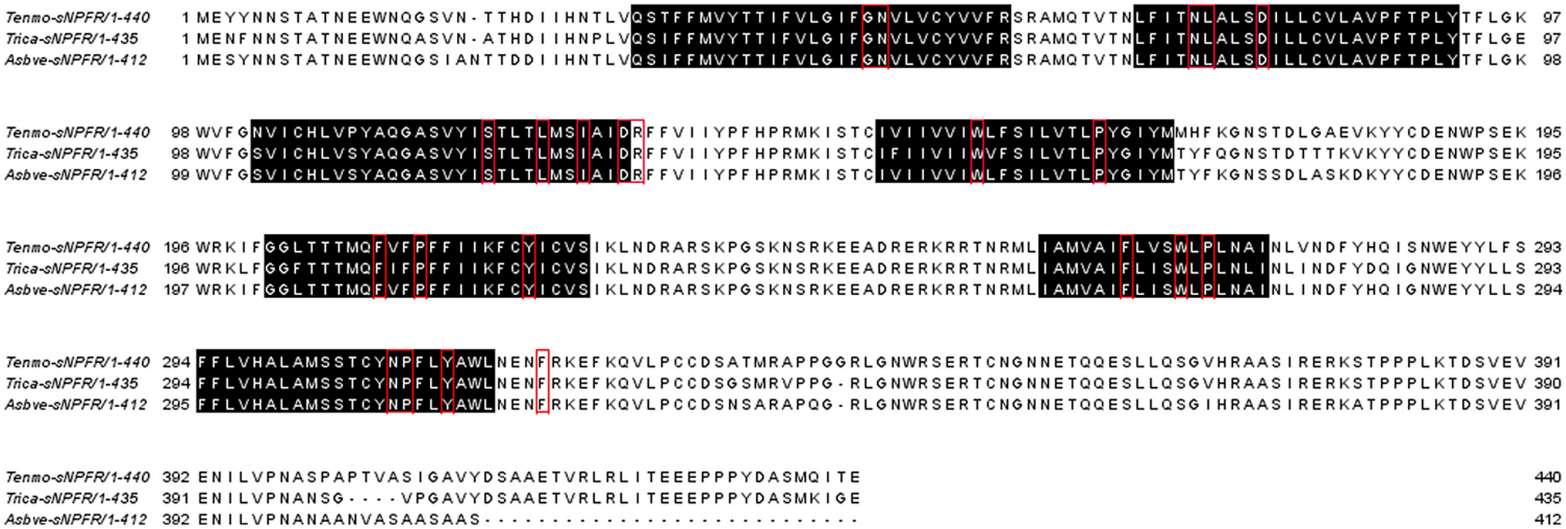
Alignment of the sNPFRs sequences from *T. molitor* (Tenmo-sNPFR), *T. castaneum* (Trica-sNPFR) and *A. verrucosus* (Asbve-sNPFR), all from the Tenebrionidae family. Predicted seven transmembrane domains are highlighted in black, whereas the typical rhodopsin-like GPCR motifs are marked in red boxes

**Fig. 2:**
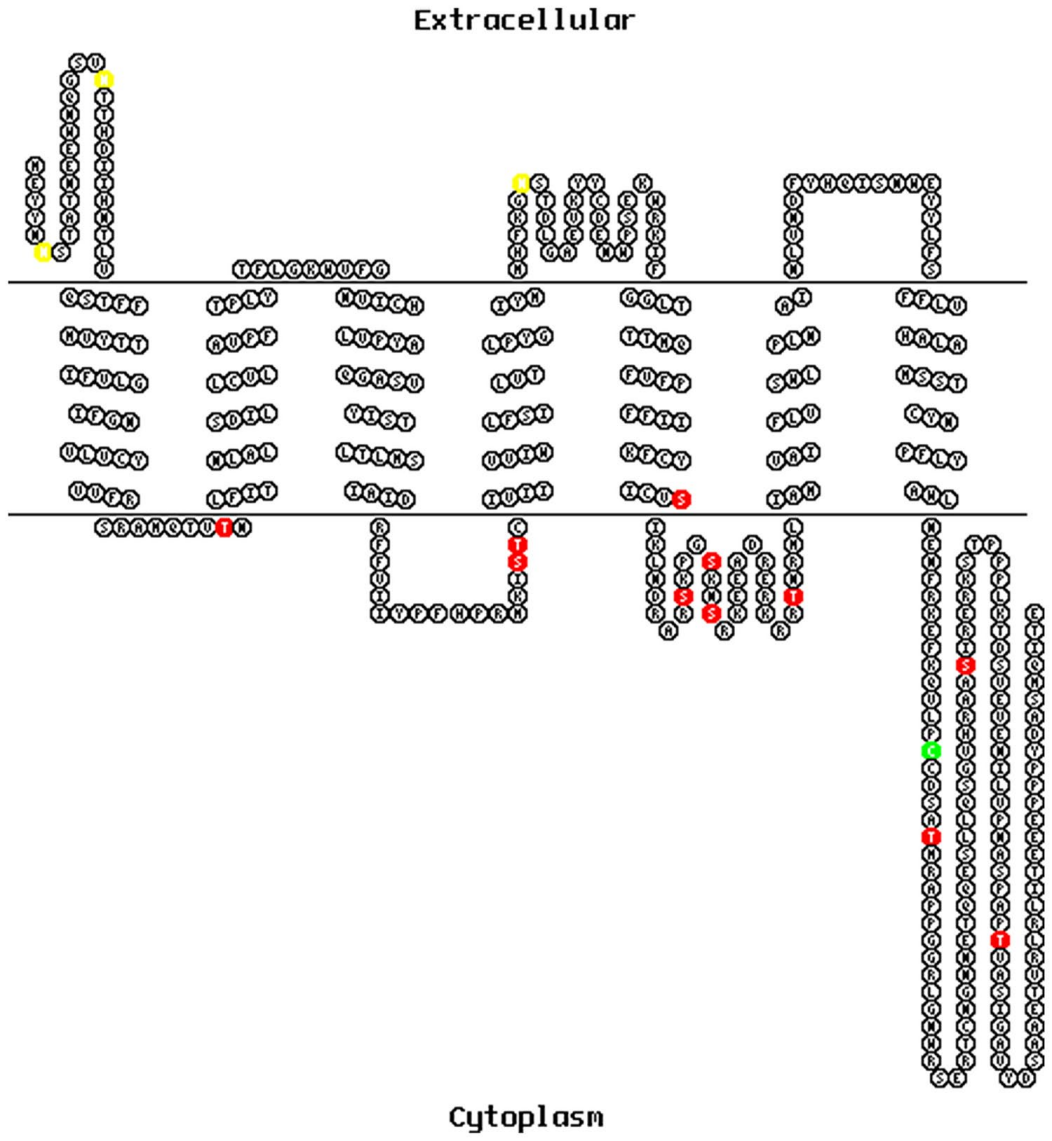
Protein structure model of *T. molitor* sNPFRs. The schematic representations indicate the orientation of the receptor protein and seven transmembrane domains. Predicted glycosylation sites are highlighted in yellow, in red putative phosphorylation sites and in green predicted palmitoylation sites

Based on the BLAST search with local database—transcriptomic assembly of *T. molitor* brain and retrocerebral complex yielded an open reading frame with 1320 bp which encodes a putative short neuropeptide F receptor (sNPFR) (Fig. 1). The Tenmo-sNPFR displays the seven transmembrane domains typical for GPCRs (Bass et al. 2014) with *N*-terminal ligand binding tail and *C*-terminal intracellular region (Fig. 2). The predicted post-translational modifications of the receptor protein include the typical glycosylation of *N*-terminal region and extracellular loops, phosphorylation by protein kinase C and cAMP and cGMP dependent kinases and palmitoylation at the *C*-terminal tail (Fig. 2). Protein sequence alignment with other tenebrionid beetles shows a very high degree of identity and similarity—91% with *T. castaneum* and 87% with *A. verrucosus* (Fig. 1). Only as expected slightly higher variability was observed in *N*-terminal and *C*-terminal regions (Fig. 1, Suppl. material 1). Practically 100% identity was observed in sequences of transmembrane helixes (Fig. 1).

The same identity was observed when the putative Tenmo-sNPFR protein sequence was aligned with sNPFRs sequences from other beetles obtained from public databases. The helixes sequences were again identical (Suppl material 1), whereas the highest variability was shown in *N*- and *C*-terminal regions and extracellular loop 2 (ECL2). The overall similarity of the Tenmo-sNPFR protein sequence to 11 Coleoptera species was also very high and never dropped below 60% (Suppl material 2). The lowest similarity was observed when Tenmo-sNPFR was compared to *A. plannipenis* sNPFR (65%), whereas the highest one outside the Tenebrionidae family was observed after comparison with *A. glabripennis* from the Cerambycidae family and *A. tumida* from the Nitidulidae family (Suppl material 2). All aligned Coleopteran sNPFR protein sequences were quite similar. The highest variability was observed again in the ECL2 between transmembrane helixes 4 and 5 (Suppl mat. 1).

### Distribution of sNPFR transcript in reproductive tissues of *T. molitor* males

**Fig. 3:**
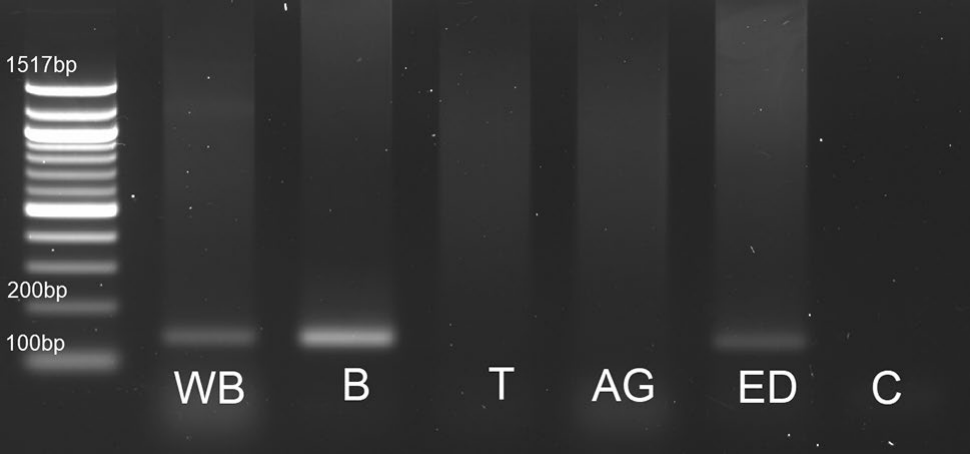
Tissue distribution of the sNPFR transcript in *T. molitor* males. *WB* whole body, *B* brain, *T* testes, *AG* accessory gland, *ED* ejaculatory duct, *C* control (H_2_O)

To check whether the peptides are able to influence the reproductive tract directly we examined the Tenmo-sNPFR tissue distribution by RT-PCR. As a positive control, we used brain tissue in which sNPFR was shown to be present in various insects (Nӓssel and Wegener 2011). Analysis of Tenmo-sNPFR transcript distribution revealed that it is present in the brain-positive control and ejaculatory duct tissue used for RNA isolation. It proves that sNPFR is not present in the testes and the accessory glands but only in the ejaculatory duct (Fig. 3). Bands intensity, despite the fact that there were not quantified, indicates that the level of the transcript may vary between tissues tested and, as expected is the highest in the brain and much lower in the ejaculatory duct.

### Changes in soluble protein concentration and dry mass in the testes

**Fig. 4:**
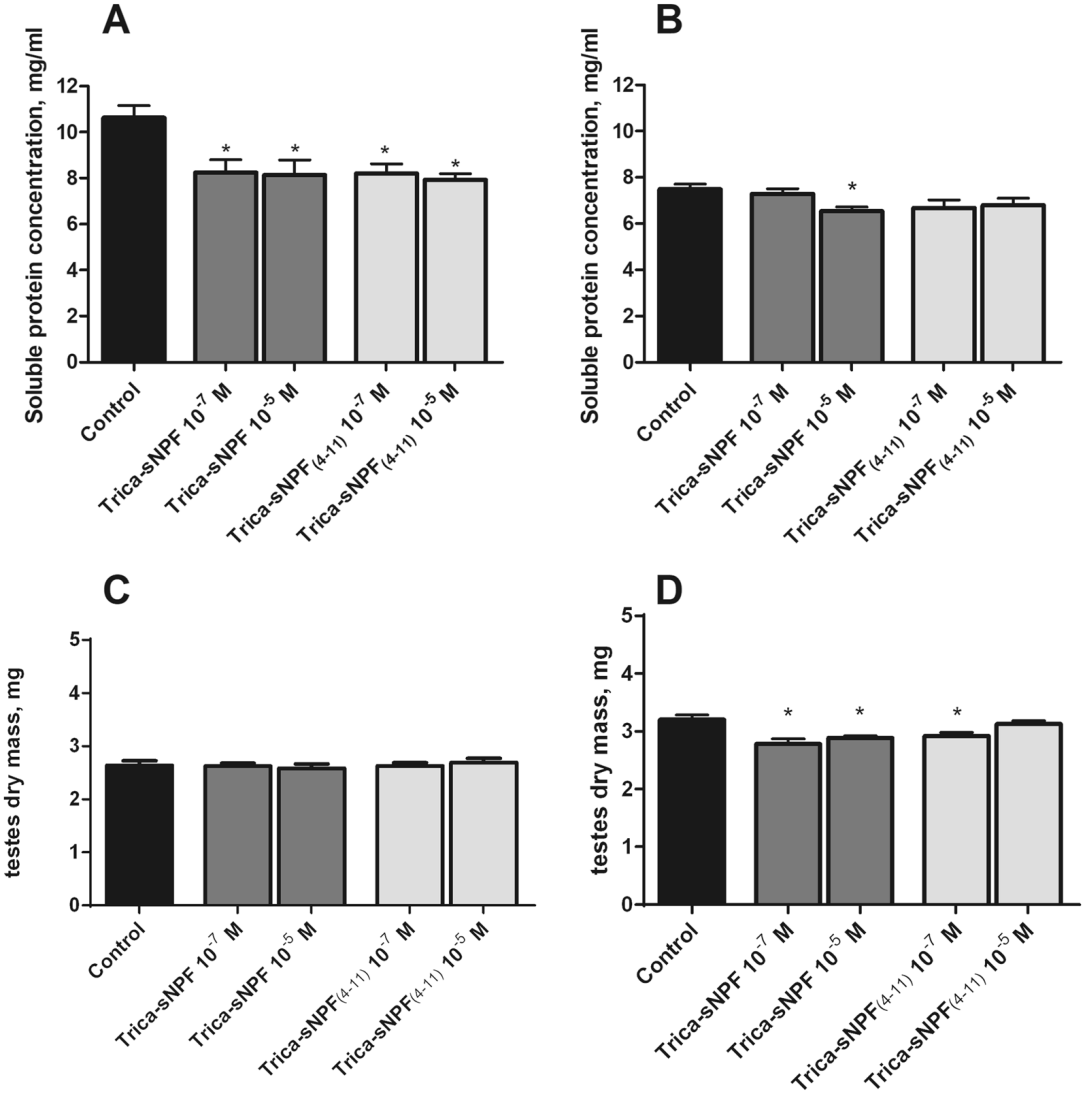
Changes in the soluble protein concentration in the testes of 4- (**a**) and 8-day-old (**b**) *T. molitor* males together with changes in the dry mass of these organs in 4- (**c**) and 8-day-old (**d**) males 24 h after peptide injection. Significant changes in relation to the control are indicated by an asterisk (Mann–Whitney test; *p* ≤ 0.05); *n* ≥ 10. Values are means ± SEM

In the supernatants prepared from the five testes isolated from 4- and 8-day-old *T. molitor* males, mean soluble protein concentration were slightly different between groups. A higher concentration was observed in younger males at approximately 10.5 mg/mL compared to older individuals at 7.5 mg/mL (Fig. 4a, b). Injections of Trica-sNPF and Trica-sNPF_4–11_ in both physiological and pharmacological concentrations decreased the level of the soluble protein fraction in 4-day-old males (Fig. 4a). The decrease was 22% and 23% after injections of 10^–7^ (Mann–Whitney test; *U* = 8; *P* = 0.0103) and 10^–5^ M (Mann–Whitney test; *U* = 12; *P* = 0.0153) of sNPF, respectively (Fig. 4a). The sNPF_4–11_ injections caused similar decrease of 22% after 10^–7^ M (Mann–Whitney test; *U* = 15; *P* = 0.0037) and 24% in 10^–5^ M (Mann–Whitney test; *U* = 6; *P* = 0.0048). The observed effects were much stronger in younger males than in 8-day-old ones (decrease by 13%). In older insects, the decrease was dose dependent and significant effects were observed only after injections of Trica-sNPF in 10^–5^ M (Mann–Whitney test; *U* = 12; *P* = 0.0241) (Fig. 4b). After injections of shorter peptide the effects were similar as for the longer one in both concentrations, however, not statistically significant (Fig. 4b) (Mann–Whitney test; *U* = 14; *P* = 0.0986 and Mann–Whitney test; *U* = 26; *P* = 0.0961).

Decreasing the soluble protein concentration was related to the slight changes in the dry mass of the testes. The dry mass of the testes in 4-day-old insects was about 2.6 mg, whereas in older individuals the average dry mass was about 3.2 mg. Injections of the tested peptides caused a decrease in the dry mass of testes isolated from 8-day-old males and the strongest effect was observed after injection of Trica-sNPF in the physiological concentration (Fig. 4d) (Mann–Whitney test; *U* = 10; *P* = 0.0056). The effect of the shorter form was only significant at lower concentration (Mann–Whitney test; *U* = 13; *P* = 0.0141). Surprisingly, in 4-day-old insects both peptides did not produced any effects neither in 10^–7^ nor 10^–5^ M concentration (Fig. 4c).

### Changes in protein concentration and dry mass in the accessory glands

To check whether short neuropeptides F might be involved in the functioning of accessory glands and thus influence the composition of the seminal fluid, we measured the soluble protein concentration in the supernatant produced from this organ and its dry mass after an application of the peptides.

**Fig. 5:**
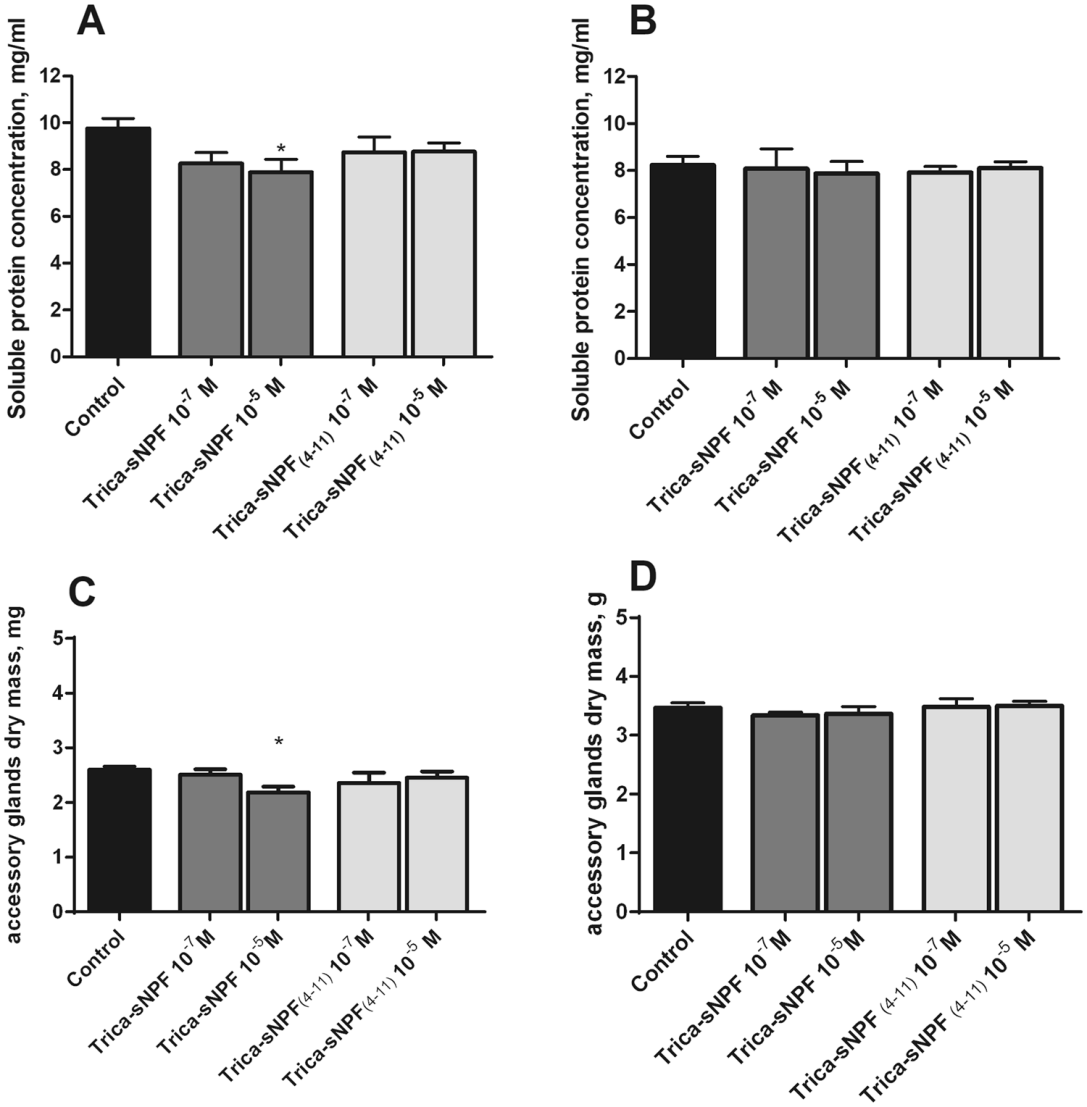
Changes in the soluble protein concentration in the accessory glands of 4- (**a**) and 8-day-old (**b**) *T. molitor* males together with changes in the dry mass of these organs in 4- (**c**) and 8-day-old (**d**) individuals 24 h after peptide injections. Significant changes in relation to the control are indicated by an asterisk (Mann–Whitney test; *p* ≤ 0.05); *n* ≥ 10. Values are means ± SE

Mean protein concentration in the supernatants obtained from 5 bean-shaped accessory glands varies between groups. In younger males, the average concentration was around 9.7 mg/mL, while in 8-day-old insects ~ 8.2 mg/mL (Fig. 5a, b). Injections of Trica-sNPF and its shorter form caused effects similar to the one observed for testes but much weaker. In 4-day-old males, the tendency of both neuropeptides to decrease the soluble protein concentration by about 10–20% depending on concentration tested was shown (Fig. 5a). The strongest effect (decrease of 22%) was observed after application of Trica-sNPF in 10^–5^ M (Mann–Whitney test; *U* = 13; *P* = 0.0203). The lower concentration of this peptide caused a decrease of 15%; however, the results were not statistically significant, so were the effects of Trica-sNPF_4–11_ injections in both concentrations (Fig. 5a). In older individuals, no significant effects were observed after injections of Trica-sNPF. Both tested concentrations were ineffective, so were both concentrations of Trica-sNPF_4–11_ (Fig. 5b).

Similar to testes, the dry mass of the accessory glands was lower in younger males (2.6 mg) and higher in older ones (3.5 mg). Injections of tested neurohormones caused dose-dependent decrease in the dry mass of the organs (Fig. 4c, d). In 4-day-old males, a decrease was observed in all tested experimental systems. The strongest significant effect (15%) was shown after injection of long sNPF in concentration 10^–5^ M (Fig. 5c) (Mann–Whitney test; *U* = 12.5; *P* = 0.0032). In 8-day-old males all tested concentrations for both peptides were ineffective.

### Total spermatocyte number

**Fig. 6:**
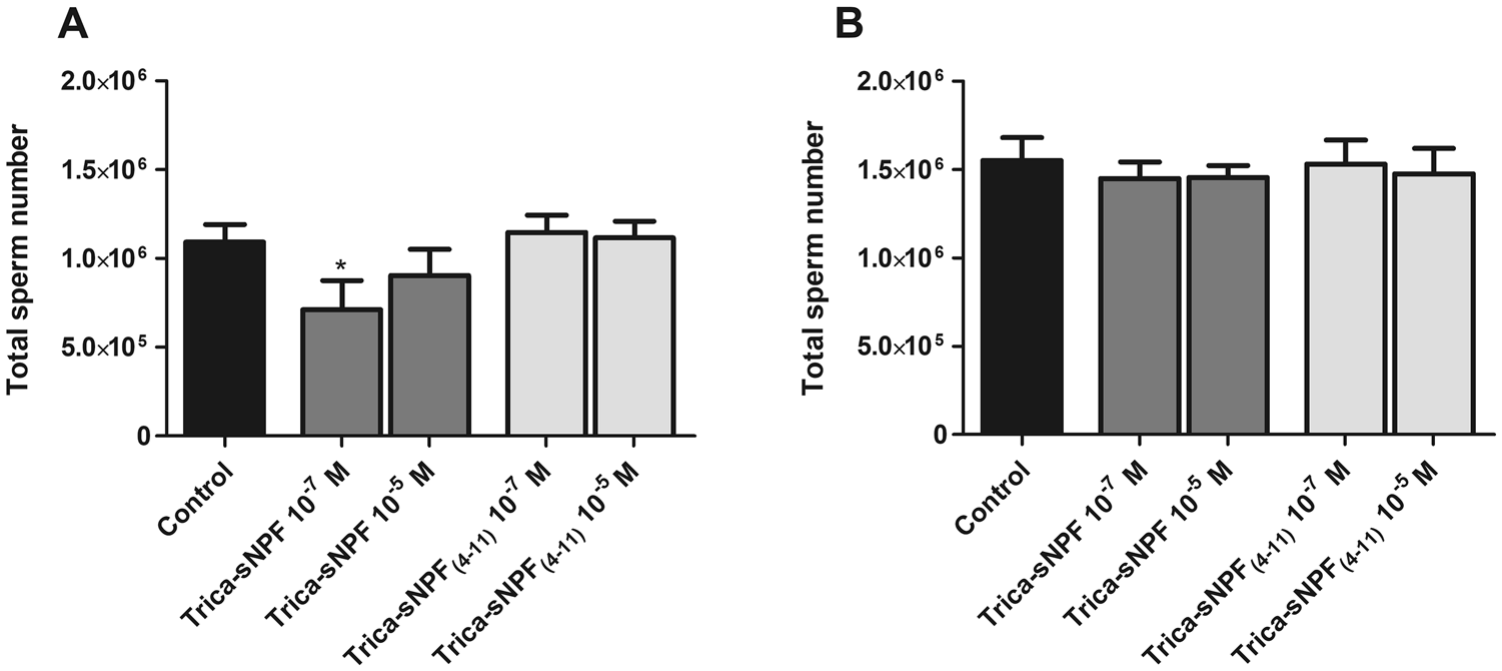
Changes in the total number of sperm cells in 4- (**a**) and 8- (**b**) day-old *T. molitor* males 24 h after injections of the tested peptides. Values are means ± SEM, and asterisks indicate significant differences (Student *t* test) relative to the control; *n* ≥ 10.

The total number of spermatocytes in 4-day-old males injected with physiological saline was ~ 1092 × 10^6^ ± 101,319 and it was lower than in 8-day-old individuals (1552 × 10^6^ ± 130,530) by half a million (Fig. 6a, b). Injections of tested peptides caused dose-dependent and age-dependent changes in the number of spermatocytes isolated from the testes.

**Fig. 7:**
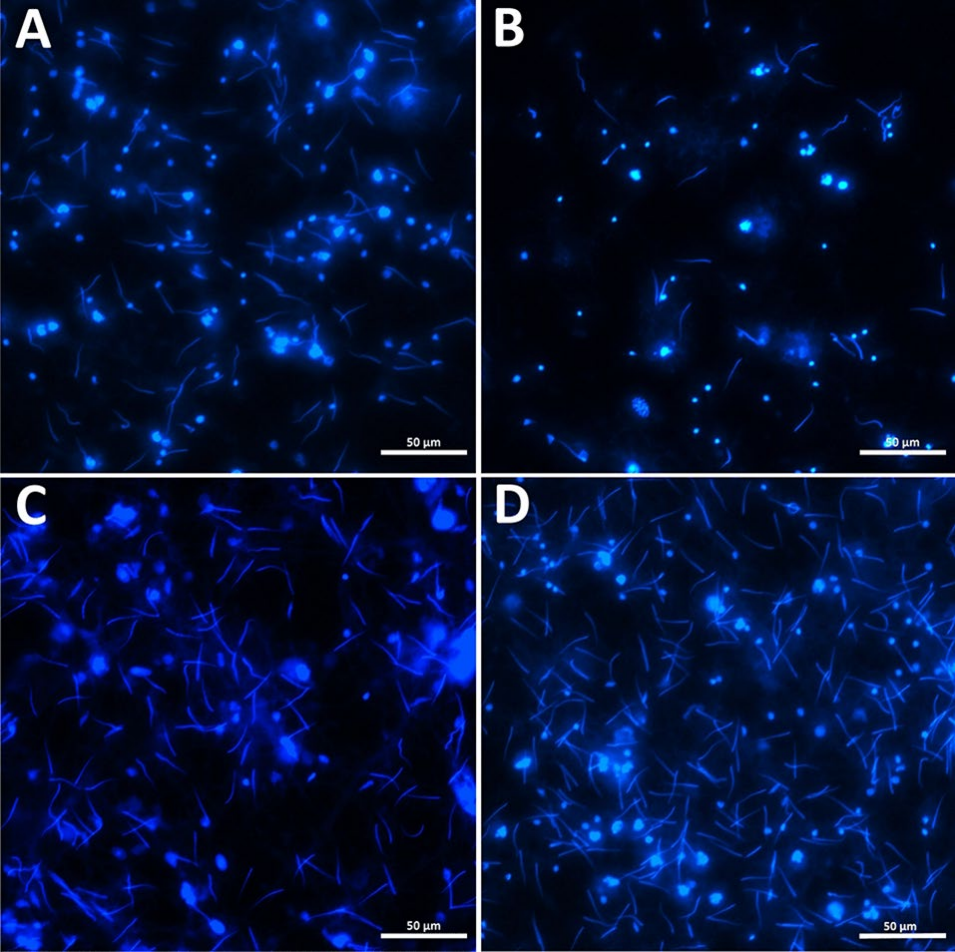
Representative microscopic photos of spermatozoid nuclei of *T. molitor* males. For visualization of spermatozoid nuclei (blue), DAPI staining was used. **a**, **b** Spermatozoid nuclei of 4-day-old individuals injected 24 h earlier with physiological saline (**a**) or Trica-sNPF at a concentration 10^–7^ M (**b**); **c**, **d** photos of spermatozoid nuclei of 8-day-old individuals 24 h after injection of physiological saline (**c**) and Trica-sNPF_(4–11)_ at a concentration 10^–7^ M (**d**). The scale bar indicates 50 µm

Injection of Trica-sNPF into younger males caused a decrease in the number of spermatocytes in both concentrations; however, only at a peptide concentration of 10^–7^ M the effect was statistically significant (Mann–Whitney test; *U* = 49.5; *P* = 0.0301). Both concentrations tested for Trica-sNPF_(4–11)_ caused no effects (Fig. 6a). In older ones, similarly to 4-day-old individuals, the slight tendency to decrease the number of spermatocytes was observed but no statistically significant differences were indicated (Figs. 6b, 7d).

### Effect of peptides on ejaculatory duct contractions in vitro

**Fig. 8:**
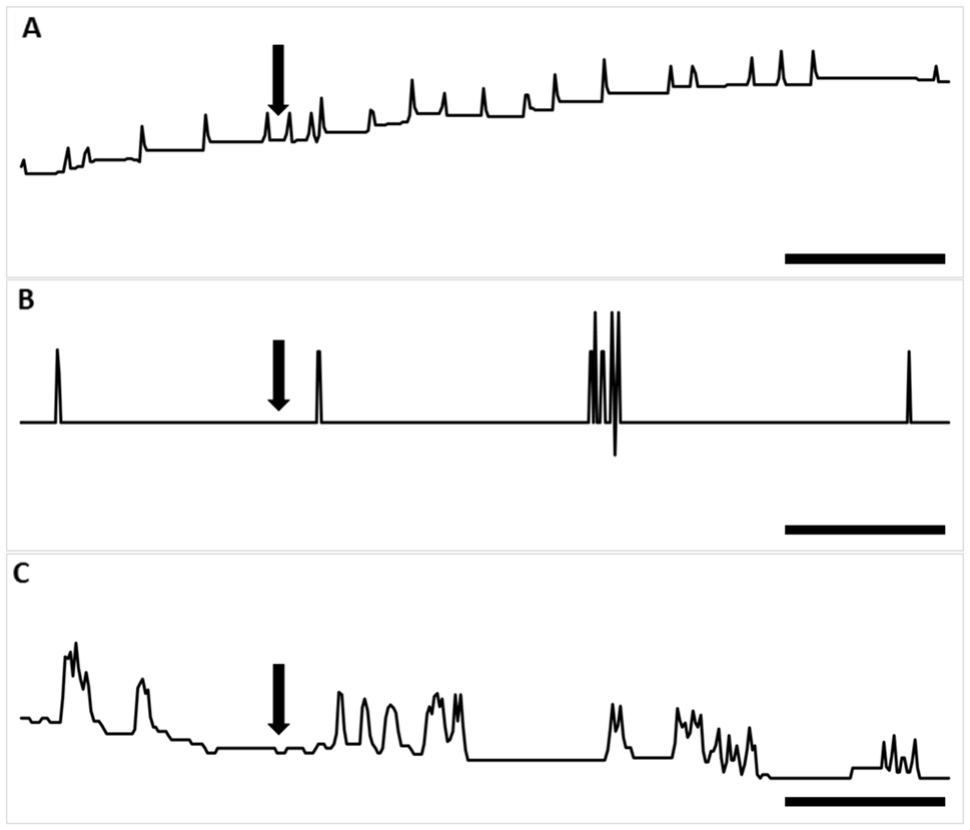
Typical responses in the contraction frequency of the *T. molitor* ejaculatory duct after the application of physiological saline (**a**), Trica-sNPF at 10^–12^ M (**b**) and Trica-sNPF_(4–11)_ at 10^−6^ M (**c**). The arrows show the time of neuropeptide application. Scales indicate 20 s of video recording

The ejaculatory duct of the *T. molitor* adult male showed irregular contractions during the perfusion with physiological saline with an average of 10 ± 9 contractions/min. Both tested peptides caused reversible myostimulatory effects on the ejaculatory duct contractions (Fig. 8).

Trica-sNPF stimulated contractions of the ejaculatory duct in the dose-dependent manner (Fig. 8a, b). Both peptides caused the strongest increase in contraction frequency (18% for sNPF and 17% for sNPF_(4–11)_ after application of the highest tested concentration 10^–5^ M when compared to control (physiological saline application) (Fig. 8a, b). All other tested concentrations caused slight myostimulatory effects (around 4% after 10^–10^ M for both peptides and around 10% after 10^–8^ M for both peptides).

**Fig. 9:**
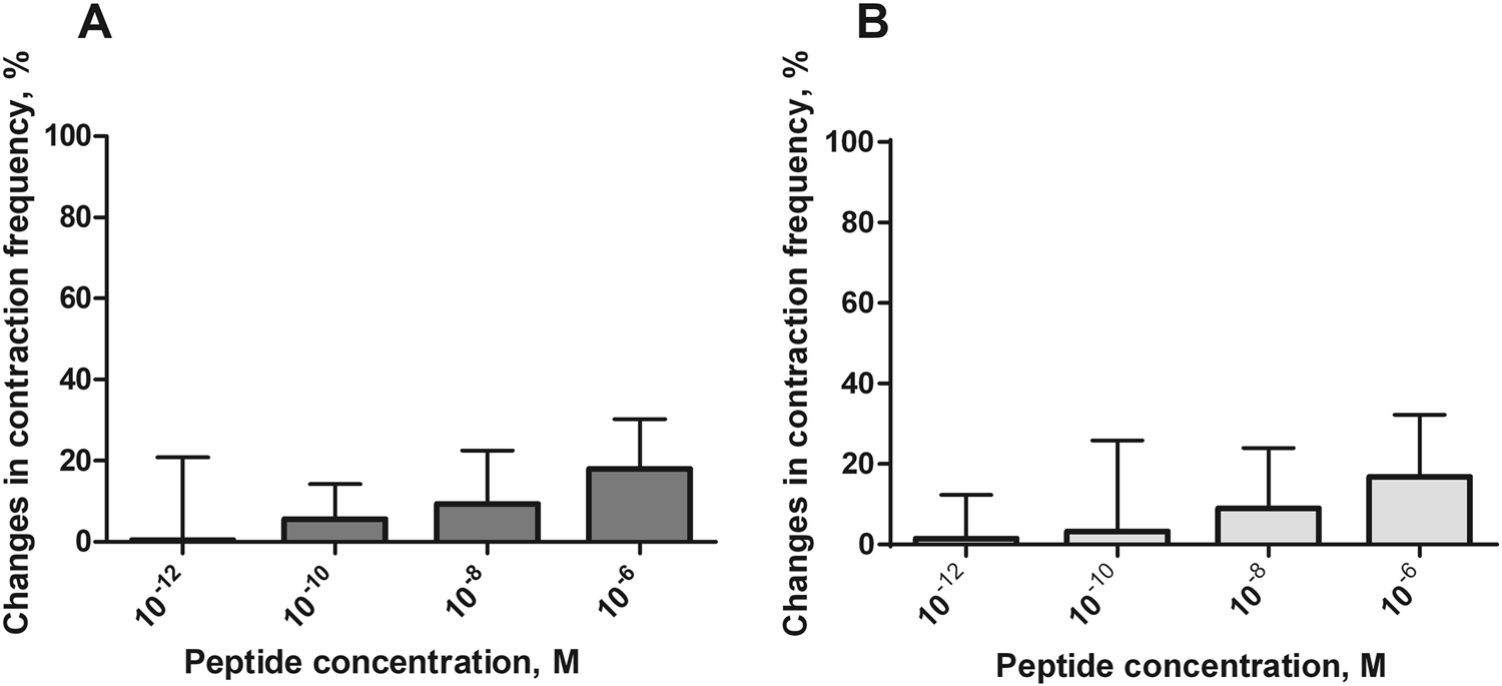
Changes in contraction frequency of ejaculatory duct after application of Trica-sNPF (**a**) and Trica-sNPF_(4–11)_ (**b**) peptides. Statistically significant results when compared to control–physiological saline application are indicated by asterisks (unpaired *t* test with Welch’s correction); *n* ≥ 15. Values are means ± SEM

Statistically significant differences from control was not observed; however, responses to the two other concentrations of both peptides are evident (Fig. 9a, b).

### Effect of peptides on males fertility

To check whether tested peptides affect fertility of males injected individuals were introduced to females. Mated females were allowed to deposit their eggs and eggs were allowed to hatch.

**Fig. 10:**
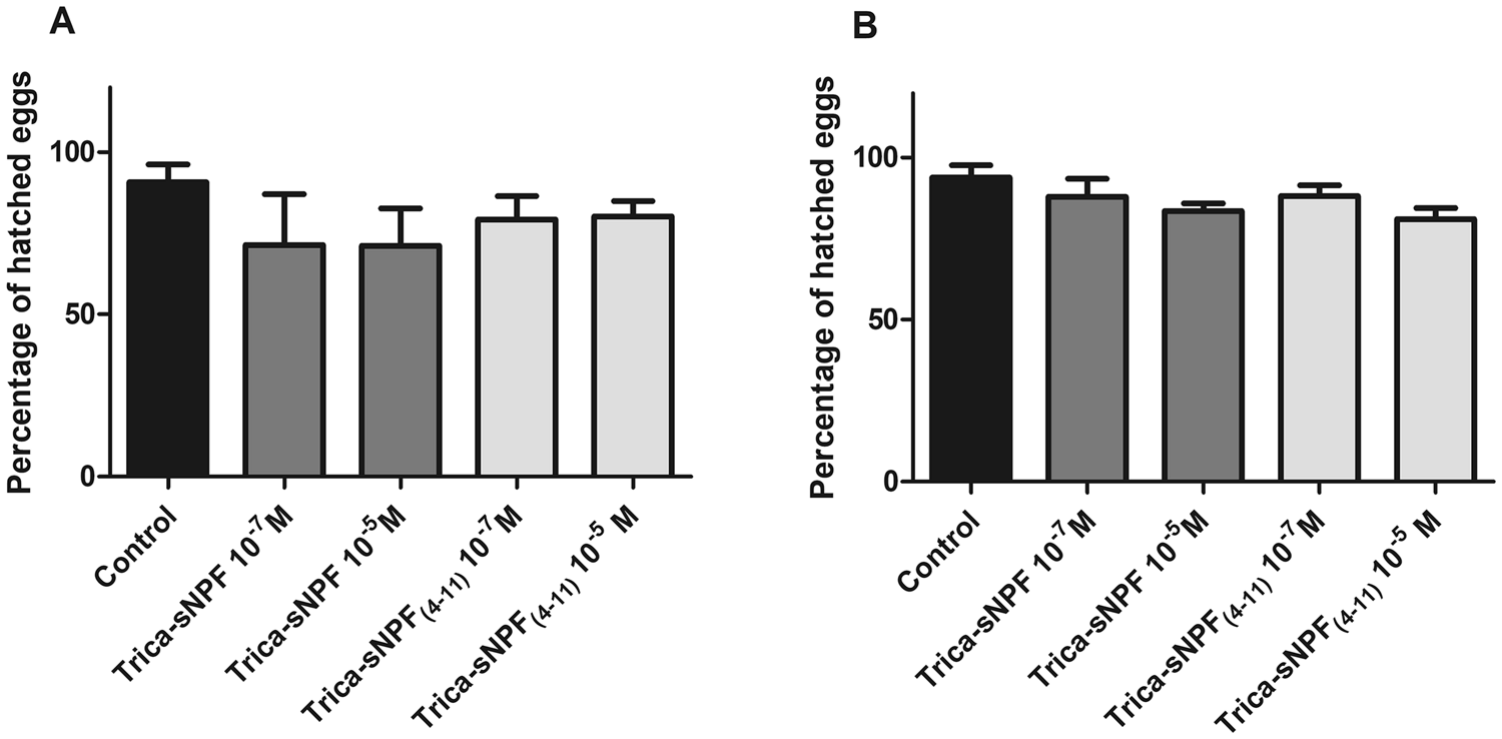
Percentage of hatched eggs laid by females introduced to 4- (**a**) and 8- (**b**) day-old *T. molitor* males 24 h after injections of the tested peptides. Values are means ± SEM, and asterisks indicate significant differences (Mann–Whitney test) relative to the control; *n* ≥ 5

Percentage of hatched eggs for both experimental variants were high—91% for 4-day-old and 94% for 8-day-old males. Injection of younger males by Trica-sNPF caused a decrease in egg hatching for 22% (Fig. 10a). The effects were not dose-dependent. Smaller decrease (12%) was observed after injections of Trica-sNPF_(4–11)_. Injection of older males caused smaller decrease in percentage of hatched eggs. The more potent for both peptides was higher tested concentration 10^–5^ M (decrease by 11% for sNPF and 14% for sNPF_(4–11)_). Higher tested concentrations caused less evident effects (Fig. 10b). Although the observed effects were not statistically significant the effects of peptides injections, especially in 4-day-old males were evident.

## Discussion

The present study expands the knowledge about the physiological role of short neuropeptides F in the largest group of insects—Coleoptera. Here, we have examined the role of sNPF in the regulation of reproductive events in the *T. molitor* beetle. We used sNPF peptide Trica-sNPF (SGRSPLRLRFa) from very closely related species *T. castaneum.* Recently published in silico predictions showed that putative Tenmo-sNPF (SSRSPLRLRFa) differs in only one amino acid (Veenstra 2019). In position 2 glycine is changed to serine thus this should be irrelevant in this study. We showed that injections of synthetic sNPF peptides affects the total sperm number, testes and accessory gland soluble protein content and the dry mass of testes. On the other hand, peptides influenced the contractility of ejaculatory duct in vitro and these effects seems to be direct and tissue-specific. The peptides acts via the putative GPCR characterized in this species. Moreover, we showed that these peptides probably might decrease the fertility of the males.

sNPF signaling has been studied in variety of insect species, including mosquitoes (Christ et al. 2018), locusts (Dillen et al. 2013), flies (Caers et al. 2016; Feng 2003) or ants (Chen and Pietrantonio 2006). In all of these species, sNPF receptor was identified and its protein sequence was analyzed. In beetles, the first sNPFR was identified in *T. castaneum* (Hauser 2008). As for most of the neuropeptides also sNPFRs belong to G-protein-coupled receptors with seven transmembrane helixes, *N*-terminal extracellular segment and the intracellular *C*-terminal tail responsible for interactions with G proteins (Hauser 2008). Moreover, a detailed analysis of sNPFR showed that all sNPFRs identified so far belong to the subfamily A rhodopsin-like GPCRs (Caers et al. 2016). Also, the receptor predicted in this study resembles this type of receptors (Suppl mat 3). It contains typical rhodopsin-like amino acid patterns in its seven transmembrane domains: GN in helix 1, NLX3-DX8P in helix 2, SX3LX2IX2DRY in helix 3, WX8P in helix 4, FX2PX7Y in helix 5, FX3WXP in helix 6 and NPX2YX6F in helix 7 (Fig. 1) (Costanzi 2012). The presence of multiple putative sites for phosphorylation, glycosylation and palmitoylation suggests post-translational modifications that may regulate the activity of Tenmo-sNPFR (Fig. 2). The bioinformatic analysis showed that based on sequence similarities receptors for the sNPFs cluster together with the receptor for prolactin-releasing peptide (PrRP) (Jekely 2013). For example, sequence similarity between Tenmo-sNPF and human PrRP is about 40% (Suppl mat 3). This suggests that sNPF may have evolved into the prolactin-releasing peptide signaling system, that also regulates feeding and has been suggested to be orthologous to sNPF (Fadda et al. 2019). PrRP is a member of RFamide peptide superfamily and in mammals its role, despite the name, is no longer associated with release of prolactin. It has been proven that this peptide in vertebrates is involved in the regulation of feeding (Kunes et al. 2016), indicating the structural and functional similarities of sNPF and PrRP between insects and mammals.

The detailed distribution of sNPFR in tissues has also been extensively studied to reveal the exact role of sNPF in insect physiology. Thus far, they have been mainly associated with regulation of feeding and metabolism (Fadda et al. 2019). The spatial distribution of the receptor does not always reflect only this function of sNPF. The receptor transcript in *Schistocerca gregaria* seems to be limited to the various parts of the nervous system (Dillen et al. 2013, 2014). However, in the flies *D. melanogaster, Glossina morsitans* and ant *Solenopsis invicta*, in addition to the nervous system, sNPFR expression was observed in a variety of peripheral tissues, such as the gut, fat body, spermatheca, ovaries and Malpighian tubules (Chintapalli et al. 2007; Lu and Pietrantonio 2011; Mertens et al. 2002). This is partially consistent with our studies. The transcript for Tenmo-sNPFR appears to be the highest in the brain, but it was also present in the ejaculatory duct. The sNPFR transcript was not detected in locust *S. gregaria* and tse-tse fly *G. morsitans* were testes and accessory glands were examined (Caers et al. 2016; Dillen et al. 2013). This proves that observed effects in these tissues might be indirect. In mosquitoes, the total abdomen tissue was analyzed so it was not possible to determine the exact spatial distribution of the receptor, but we cannot exclude that it is present in the male reproductive tract (Garczynski et al. 2007).

sNPFs in insects can act as neuromodulators or hormones (Nӓssel and Wegener 2011). Their major role in insects seems to be the regulation of feeding behavior, both as stimulatory or inhibitory factors (Fadda et al. 2019). However, they also regulate body size due to the interaction with insulin-like peptides (Fadda et al. 2019) and are involved in the control of circadian rhythm interacting with pigment dispersing factor (Vecsey et al. 2014). In *B. mori,* it was also shown that sNPF peptides might be involved in the regulation of juvenile hormones (JH) biosynthesis (Kaneko and Hiruma 2014). sNPFs interplaying with other hormones, such as allatostatin C and dopamine, decrease the JH level and thus initiate pupal metamorphosis (Kaneko and Hiruma 2014). This all indicate that these peptides regulate insect development. In beetles, sNPFs were also shown to regulate developmental processes. When identifying of one of the first sNPFs in *L. decemlineata* it was shown that Lepde-sNPF-I and Lepde-sNPF-II are present only in non diapausing insects, whereas in diapausing ones they are absent (Huybrechts et al. 2004). Also in *T. molitor,* Lepde-sNPF-I regulates development, including pupation (Marciniak et al. 2013). Taken together all above results it is possible that sNPFs are involved in the development and reproduction in insects; however, the effects might be direct via the receptors or indirect via other hormones. Another possibility is that sNPF are involved in regulation of different processes such as circadian clock or feeding (Nӓssel and Wegener 2011) and thus affect reproduction processes in males. This is proved here, because the observed gonadoinhibitory effects were much stronger in younger individuals. Overall we showed that sNPF decrease the fertility of males. This is connected to the lower sperm number which is probably the effect of decrease of protein content in the testes. This may suggest that spermatogenesis in tested insects was interrupted; however, without detailed proteomic analysis, we cannot confirm it. In *D. melanogaster* it was shown that testes contain proteins crucial for sperm development (Takemori and Yamamoto 2009). This might be the case in beetles as well.

Another aspect of male reproduction which is important for successful reproduction is the composition of seminal fluid. It is secreted by testes, seminal vesicles and male accessory gland and responsible for protection and nourishment of sperms increasing their chance for successful fertilization (Xu et al. 2013). Here we showed that sNPF also decreases mainly the dry mass of the accessory gland. This indicates that the seminal fluid composition might be changed and thus this could also influence fertility of the males. The detailed proteomic study would be helpful to confirm this hypothesis. On the other hand studies performed on *D. melanogaster* showed that JH affect the protein synthesis in male accessory gland (Wilson et al. 2003). If sNPF affect JH levels as written above, it will indirectly regulate functioning of this organ. Secretion of accessory gland is crucial for reproduction success also for females (Lenaerts et al. 2019).

Previously, we showed that other peptide hormones Neb-collostatin and Neb-TMOF regulate the functioning of the male reproductive system (Marciniak et al. 2017). These peptides in the age-dependent manner increased the concentration of soluble protein in the testes and the dry mass in the testes of the *T. molitor*. They also significantly changed the protein profiles of the testes (Marciniak et al. 2017). A member of sNPF family Ledpe-sNPF-I was also included in these studies and showed different effects to Trica-sNPF in this paper. In general, Lepde-sNPF-I injections increased the protein concentration and dry mass of the testes. In case of soluble protein concentration, the stronger effect was observed at a lower concentration (Marciniak et al. 2017). Lepde-sNPF-I increased also the number of sperm cells in 4-day-old males. Trica-sNPF tested here in general decreased the soluble protein concentration and dry mass of the testes. These different effects are probably due to major variations in the Lepde-NPF-I (ARGPQLRLRFa) and Trica-sNPF (SGRSPSLRLRFa) peptide structure. These results confirm partially our studies. We showed that the observed effects are stronger after injection of longer Trica-sNPF when compared to shorter Trica-sNPF_(4–11)_ peptide. This is the evidence that *N*-terminal part of the molecule is crucial for its activity. These results are in agreement with similar studies performed on mosquitoes, where authors showed that sNPF_(4–11)_ was less efficient in receptor activation (Christ et al. 2018). Detailed modeling or structure–activity studies are needed to confirm this hypothesis. This supposition was confirmed with FMRFamide related family of peptides (FaRPs), another member of RFamide peptide superfamily. Structure–activity studies performed on *D. melanogaster* with different FaRPs analogues showed that the C-terminal sequence is crucial for docking to the receptor but N-terminal sequence confer docking and activity (Maynard et al. 2013). Important role of N-terminus was also showed with myosuppressin (TDVDHVFLRFa) when studied its receptor interactions in the insect heart (Leander et al. 2015).

Other studies have also shown that neuropeptides can alter the functioning of the male reproductive system. Van Wielendale et al. (2013) showed that injections of trNPF (a member of the long neuropeptide F family) increase the weight of the testes and seminal vesicles in locust *S. gregaria* (Van Wielendaele et al. 2013). This has also been proven in RNAi experiments (Van Wielendaele et al. 2013). This may suggest that the NPF family, is involved in the regulation of spermatogenesis.

The most widely studied tissue in the male reproductive system in terms of neuropeptidergic control is the ejaculatory duct. It is now know that the regulation of contractility of this organ is extremely complex. Various studies carried out so far have shown that the muscles of the ejaculatory duct are affected by allatostatins, proctolin, pyrokinins, myosuppressins, sulfakinins or NVP-like peptides (Marciniak et al. 2011, 2012; Marciniak and Rosinski 2010; Rankin et al. 2009). It was also shown that sNPFs affect the ejaculatory duct (Marciniak et al. 2017). Ledpe-sNPF-I stimulated the contractility of the ejaculatory duct of *T. molitor* in a dose-dependent manner; however, when using the low (10^−12^ M) and high (10^−5^ M) peptide concentrations, the frequency of contractions decreased (Marciniak et al. 2017). Again, these results are opposite to those obtained here. In our studies, longer peptide Trica-sNPF increased the contraction frequency of the ejaculatory duct in higher concentrations. It is again probably due to *N*-terminal differences in amino acid sequence (see above). The shorter form of the peptide Trica-sNPF_(4–11)_ stimulated ejaculatory duct muscles also in higher concentrations, however, with slightly less efficiency. The same situation was observed in mosquitoes (Christ et al. 2018). This might be because of importance of *N*-terminal sequence to the proper interaction with the receptor. Shorter form (SPSLRLRFa) without first three amino acids might slightly differentially interact with the receptor thus is active only in higher concentrations. Detailed molecular modeling studies are needed to reveal this assumptions.

To summarize, here we showed that sNPFs are involved in the regulation of reproductive events, affecting various reproductive tissues, including testes. The effects seem to be partially direct due to the presence of the receptor in ejaculatory duct (Fig. 3). Of course, the overall effect is caused by indirect activity that is connected to the interplay between sNPF and many other neuropeptides/hormones (Fadda et al. 2019; Kaneko and Hiruma 2014). Probably a complete neuropeptide network which regulates feeding, growth and development and to date includes insulin-like peptides (ILPs), allatostatin C (ASTC), pigment dispersing factors (PDFs) should be extended also to sNPFs.

## Electronic supplementary material

Below is the link to the electronic supplementary material.

Supplementary material 1 (PDF 448 kb)

Supplementary material 2 (PDF 226 kb)

Supplementary material 3 (PDF 314 kb)
