# Correction to: Short neuropeptide F signaling regulates functioning of male reproductive system in *Tenebrio molitor* beetle

**DOI:** 10.1007/s00360-023-01491-8

**Published:** 2023-05-09

**Authors:** Paweł Marciniak, Arkadiusz Urbański, Jan Lubawy, Monika Szymczak, Joanna Pacholska-Bogalska, Szymon Chowański, Mariola Kuczer, Grzegorz Rosiński

**Affiliations:** 1 Department of Animal Physiology and Development, Adam Mickiewicz University, Poznań, Uniwersytetu Poznańskiego 6 Street, 61-614 Poznan, Poland; 2 HiProMine S.A, Poznańska 8 Street, 62-023 Robakowo, Poland; 3 Faculty of Chemistry, University of Wrocław, F. Foliot-Curie 14 Street, 50-383 Wroclaw, Poland

**Correction to: Journal of Comparative Physiology B (2020) 190:521–534** 10.1007/s00360-020-01296-z

The Fig. 3 of the original publication is wrong and replaced by a new version.

The Correct figure is given below:
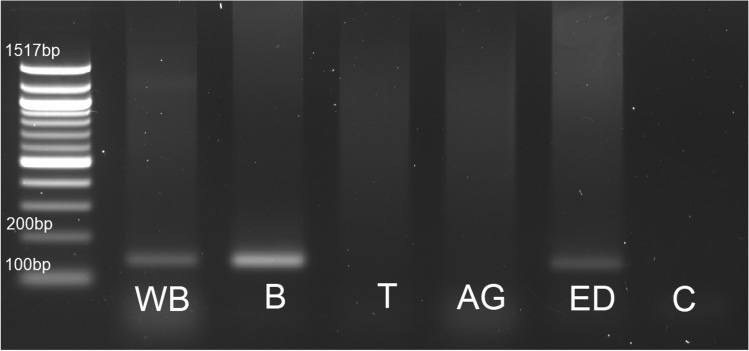


The original article has been corrected.

