# Supplementary material for: Short neuropeptide F signaling regulates functioning of male reproductive system in Tenebrio molitor beetle
Source: J Comp Physiol B. 2020 Aug 4;190(5):521–34. doi: 10.1007/s00360-020-01296-z (PMC7441091; doi:10.1007/s00360-020-01296-z)

Supplementary material 1

Sequence alignment of beetles sNPFRs. Identical and conserved amino acids across sequences are color coded in dark and light blue, respectively. Predicted transmembrane domains underlined in red.

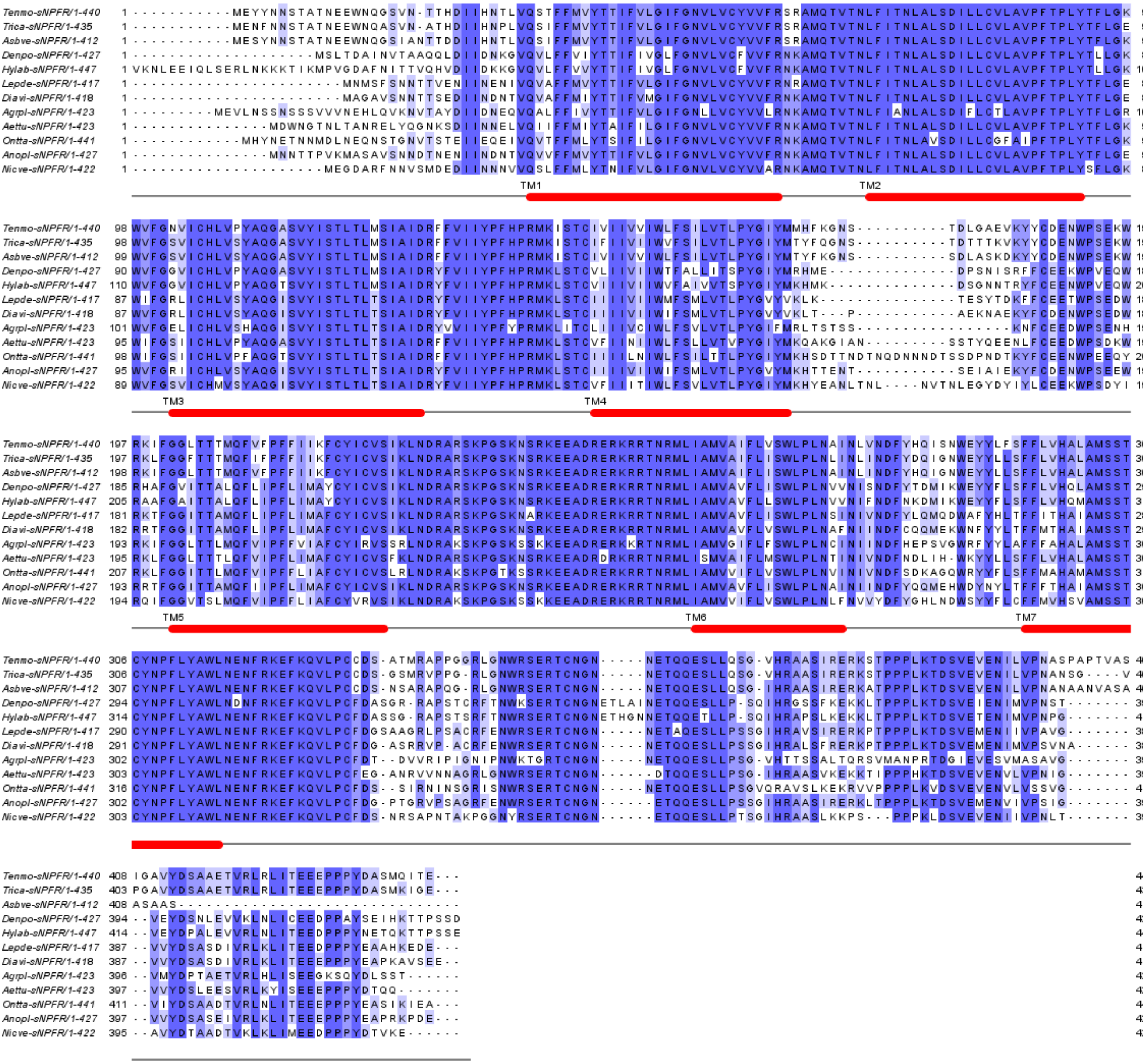

Supplement: Supplementary file 1 — Supplementary material 1 (PDF 448 kb) [file 360_2020_1296_MOESM1_ESM.pdf]
