# Supplementary material for: Short neuropeptide F signaling regulates functioning of male reproductive system in Tenebrio molitor beetle
Source: J Comp Physiol B. 2020 Aug 4;190(5):521–34. doi: 10.1007/s00360-020-01296-z (PMC7441091; doi:10.1007/s00360-020-01296-z)
Supplement: Supplementary file 2 — Supplementary material 2 (PDF 226 kb) [file 360_2020_1296_MOESM2_ESM.pdf]

Supplementary material 2

	Tenmo-sNPFR	Trica-sNPFR	Asbve-sNPFR	Denpo-sNPFR	Hylab-sNPFR	Lepde-sNPFR	Diavi-sNPFR	Agrpl-sNPFR	Aettu-sNPFR	Ontta-sNPFR	Anopl-sNPFR	Nicve-sNPFR
Tenmo-sNPFR	100%	91%	86%	67%	66%	72%	72%	65%	74%	69%	75%	68%
Trica-sNPFR	91%	100%	85%	68%	67%	74%	74%	65%	74%	69%	76%	68%
Asbve-sNPFR	86%	85%	100%	64%	62%	68%	69%	62%	71%	64%	70%	65%
Denpo-sNPFR	67%	68%	64%	100%	84%	74%	73%	60%	70%	63%	72%	66%
Hylab-sNPFR	66%	67%	62%	84%	100%	70%	70%	59%	67%	63%	70%	63%
Lepde-sNPFR	72%	74%	68%	74%	70%	100%	88%	65%	72%	69%	86%	71%
Diavi-sNPFR	72%	74%	69%	73%	70%	88%	100%	65%	73%	69%	88%	71%
Agrpl-sNPFR	65%	65%	62%	60%	59%	65%	65%	100%	65%	66%	67%	64%
Aettu-sNPFR	74%	74%	71%	70%	67%	72%	73%	65%	100%	69%	75%	70%
Ontta-sNPFR	69%	69%	64%	63%	63%	69%	69%	66%	69%	100%	70%	67%
Anopl-sNPFR	75%	76%	70%	72%	70%	86%	88%	67%	75%	70%	100%	70%
Nicve-sNPFR	68%	68%	65%	66%	63%	71%	71%	64%	70%	67%	70%	100%

Distance matrix based on sNPFRs alignment in beetles.
