# Supplementary material for: Short neuropeptide F signaling regulates functioning of male reproductive system in Tenebrio molitor beetle
Source: J Comp Physiol B. 2020 Aug 4;190(5):521–34. doi: 10.1007/s00360-020-01296-z (PMC7441091; doi:10.1007/s00360-020-01296-z)

Supplementary material 3

Sequence alignment of chosen insects sNPFRs and prolactin receptor. Identical and conserved amino acids across sequences are color coded in dark and light blue, respectively. Predicted transmembrane domains underlined in red.

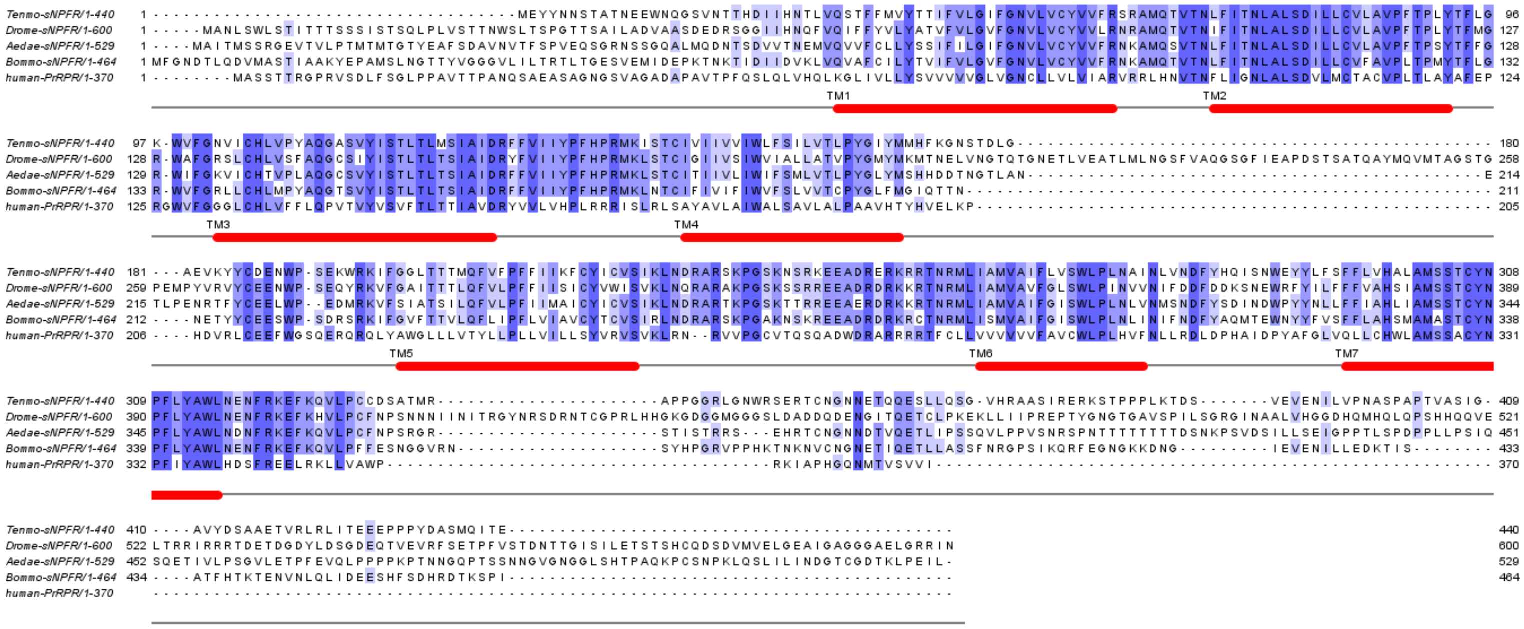

Supplement: Supplementary file 3 — Supplementary material 3 (PDF 314 kb) [file 360_2020_1296_MOESM3_ESM.pdf]
